# 63 Immunomodulators Reverse Immune Dysfunction ex-vivo After Pediatric Thermal Injury

**DOI:** 10.1093/jbcr/iraf019.063

**Published:** 2025-04-01

**Authors:** Julia Penatzer, Gabrielle Kostur, Dana Schwartz, Renata Fabia, Mark Hall, Rajan Thakkar

**Affiliations:** Burn Center at Nationwide Children’s Hospital; Nationwide Children’s Hospital; Nationwide Children’s Hospital; Nationwide Children’s Hospital; Nationwide Children’s Hospital; Nationwide Children’s Hospital

## Abstract

**Introduction:**

Pediatric thermal injury induces a heightened inflammatory response and immune dysfunction, which is associated with adverse clinical outcomes (e.g., infections). Specifically, burns with ≥20% total body surface area or ≥5% full thickness in pediatric patients, results in immune suppression and are most at risk to develop subsequent infections. As such, immunomodulating therapeutics have been of great interest to augment the immune response following thermal injury. Our hypothesis was that immune suppression after pediatric thermal injury is reversible ex-vivo using immunomodulators.

**Methods:**

Pediatric burn patients from a single, ABA-verified pediatric burn center with acute thermal injury were included. Blood samples were taken within the first week after injury. Within 1 h of collection, 50 µL of heparinized whole blood was added to two tubes of lipopolysaccharide (LPS) stimulation reagent (500 pg/mL) one of which contained granulocyte macrophage colony-stimulating factor (GM-CSF; 10 ng). The samples were incubated for 4 h at 37°C. Two additional tubes containing phytohemagglutinin (PHA; 10 μg/mL), one of which held Varlilumab (10 ng; an anti-CD27 antibody), was also incubated with 50 µL heparinized whole blood for 24 hours at 37°C. After incubation, the samples were centrifuged, and the supernatants were collected for batch cytokine analysis. A Mann–Whitney test was conducted to determine differences between burn patients who go on to develop a nosocomial infection relative to burn patients who did not. A Wilcoxon matched pairs signed rank test was performed to identify differences in ex-vivo cytokine production between samples incubated with the immunomodulators relative to samples that did not.

**Results:**

Pediatric burn patients who went on to develop a nosocomial infection displayed a decrease in both LPS and PHA induced cytokine production compared to burn patients who recovered without infection. The addition of GM-CSF (N=25) significantly increased ex-vivo LPS-induced tumor necrosis factor alpha compared to samples that did not receive GM-CSF (p=0.0157). Varlilumab (N=10) also significantly increased PHA induced interleukin-4 (p=0.0156) and interleukin-10 (p=0.0059) cytokine production in pediatric burn patients.

**Conclusions:**

The results of our studies provide early evidence that immunomodulators may enhance immune function early after pediatric burn injury and prior to the development of nosocomial infection.

**Applicability of Research to Practice:**

Based on these early studies, there may be an opportunity to reverse immune dysfunction using GM-CSF and/or CD27 specifically in patients at highest risk for infection identified through standard burn severity as well as ex-vivo induced cytokine production measurements.

**Funding for the Study:**

NIH-National Institute of General Medical Sciences

